# Sigma receptors and neurological disorders

**DOI:** 10.1007/s43440-021-00310-7

**Published:** 2021-08-05

**Authors:** Agnieszka Piechal, Alicja Jakimiuk, Dagmara Mirowska-Guzel

**Affiliations:** 1 Department of Experimental and Clinical Pharmacology, Centre for Preclinical Research and Technology CePT, Medical University of Warsaw, Banacha 1B, 02-097 Warsaw, Poland; 2 Second Department of Neurology, Institute of Psychiatry and Neurology, Sobieskiego 9, 02-957 Warsaw, Poland

**Keywords:** Sigma receptors, Sigma receptors agonists, Sigma receptors antagonists, Central nervous system, Neurological disorders

## Abstract

Sigma receptors were identified relatively recently, and their presence has been confirmed in the central nervous system and peripheral organs. Changes in sigma receptor function or expression may be involved in neurological diseases, and thus sigma receptors represent a potential target for treating central nervous system disorders. Many substances that are ligands for sigma receptors are widely used in therapies for neurological disorders. In the present review, we discuss the roles of sigma receptors, especially in the central nervous system disorders, and related therapies.

**Graphic abstract:**

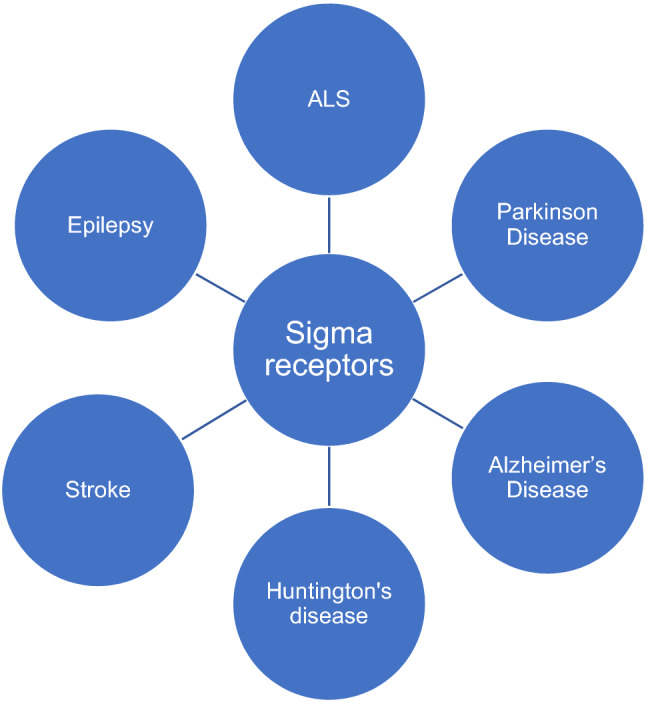

## Introduction

Sigma receptors constitute a relatively new, and not yet fully understood, type of receptor. They were first described in 1976 by Martin et al. [1]. These receptors had previously been considered a type of opioid receptor but were classified as a separate group due to their negligible affinities for naloxone and naltrexone. Sigma receptors were initially detected in the central nervous system (CNS), and further research revealed their presence in peripheral organs.

Notably, sigma receptors play significant roles in tumor cell lines of various tissues. Sigma receptor expression is high in rapidly proliferating cells and decreased among inactive tumor cells [2]. In the early 1990s, two subtypes of sigma receptors were distinguished: sigma 1 (S1R) and sigma 2 (S2R). It has been proposed that these receptors may form a potential target for the diagnosis and therapy of cancer and central nervous system (CNS) disorders [3].

PubMed lists nearly 5000 published articles on sigma receptors. In the present work, we aimed to review the literature regarding the structure and function of individual sigma receptor subtypes, with particular emphasis on their roles in the CNS and possible therapies involving compounds that act on these receptors.

### The structure and role of S1R

S1R is unlike any traditional receptor. It was first cloned in 1996, and it is a long protein (223 amino acids; 29 kDA) that is not homologous to any other known mammalian protein [4]. The gene for S1R is located on band p13 of chromosome 9. S1R exhibits a trimeric structure, with each receptor in the trimer having a single transmembrane domain that anchors it to the cytosolic side of the endoplasmic reticulum. S1R has also been detected in the nuclear and cytoplasmic membranes [5, 6]. This receptor is widely distributed throughout the CNS, as well as in the kidneys, lungs, liver, reproductive system, and tissues of the immune system. It has been suggested that the hallucinogenic compound *N*,*N*-dimethyltryptamine (DMT) may act as an endogenous S1R ligand [7]. Other exogenous ligands constitute a variety of compounds belonging to numerous drug classes, including antipsychotics (haloperidol), benzomorphan derivatives (dextromethorphan and pentazocine), antidepressants (fluvoxamine), steroids (progesterone), calcium channel blockers (verapamil and emopamil), antihistamines (chlorphenamine), antifungal drugs (fenpropimorph and tridemorph), antiestrogens (tamoxifen), and addictive compounds (methamphetamine, cocaine, and *N*,*N*-dimethyltryptamine) [8] (Table 1).

**Table 1:** Agonists and antagonist S1R

Agonist	Antagonist
Fluoxetine	Haloperidol
Fluvoxamine	E-5842 (4-(4-fluorophenyl)-1,2,3,6-tetrahydro-1-[4-(1,2,4-triazol-1-il)bu tyl]pyridine citrate)
Sertraline	BMY-14802
Escitalopram	Piperazine
Citalopram	Rimcazole (BW234U)
Donepezil	BD-1047 (*N*-(2-(3,4-dichlorophenyl)-*N*-methyl-2-(dimethylamino)ethylamine)
Ifenprodil	BD 1063 (1-(2-(3,4-dichlorophenyl)ethyl)-4-methylpiperazine)
Dehydroepiandrosterone (DHEA)	DuP734 (1-(cyclopropylmethyl)-4-(2′-(4″-fluorophenyl)-2′- oxoethyl)-piperidine HBr)
Pregnenolon	Progesterone
Igmesine (JO-1784)	RC106
Amitriptyline	NPC-16377 (6-[6-(4-Hydroxypiperidinyl)hexyloxy]-3-methylflavone HCI)
Memantine	Panamesine
PRE-084 (2-(4-morpholino)ethyl-1-phenylcyclohexane-1-carboxylate)	NE-100 (4-Methoxy-3-(2-phenylethoxy)- *N*,*N*-dipropylbenzeneethaneamine)
( +)-Pentazocine	Verapamil
Dextromethorphan	Phenylpropyloxyethelene
( +)-SKF 10,047 (( +)-*N*-allylnormetazocine)	MS-377 ((R)-( +)-1-(4-chlorophenyl)-3-[4-(2-methoxyethyl)piperazin-1-yl]methyl-2-pyrrolidinone l-tartrate)
PPBP (4-phenyl-1-(4-phenylbutyl)piperidine)	BD-1008 (*N*-[2-(3,4-dichlorophenyl)ethyl]-*N*-methyl-2-(1-pyrrolidinyl)ethylamine)
3-PPP (*N*-n-propyl-3-(3-hydroxyphenyl)piperidine)	BD-1063 (1-[2-(3,4-dichlorophenyl)ethyl]-4-methylpiperazine)
AF710B (1-(2,8-Dimethyl-1-thia-3,8-diazaspiro(4,5)dec-3-yl)-3-(1H-indol-3-yl)propan-1-one)	BD-1067 (*N*-[2-(3,4-dichlorophenyl)ethyl]-*N*-ethyl-1-pyrrolidineethanamine)
BD-737	LR-132 (( +)-3,4-dichloro-*N*-[(1R,2S)-2-(1-pyrrolidinyl)cyclohexyl]benzeneethanamine)
OPC-14523	LR-172 (*N*-[2-(3,4-dichlorophenyl)ethyl]-*N*-methyl-2-(1-homopiperidinyl)eth ylamine)
SA 4503, cutamesine (1-(3,4-dimethoxyphenethyl)-4-(3-phenylpropyl) piperazine)	UMB-101
DMT (*N*,*N*-dimethyltryptamine)	YZ-069 (*N*-phenylpropyl-*N*′-(3,4-dichlorophenethyl)piperazine0
DTG (1,3-di-o-tolylguanidyne	YZ-185 (*N*-Phenylpropyl-*N*′-(3-methoxyphenethyl)piperazine)
BD-1031 (1-[2-(3,4-dichlorophenyl)ethyl]-4-methylpiperazine)	MR309 (E-52862)
BD-1052 (*N*-[2-(3,4-dichlorophenyl)ethyl]-*N*-2-propen-1-yl-1-pyrrolidineethanamine)	AC-927 (1-(2-phenylethyl)piperidine)
Dimemorfan	
Pridopidine	
Pentoxyverin	
Methamphetamine	
MDMA (3,4-methylenedioxymethamphetamine)	
Cocaine	
ANAVEX2-73 (blarcamesine)	
Edonerpic	

As multifunctional transmembrane proteins found in the endoplasmic reticulum (ER) membrane, especially in its mitochondria-associated ER-membrane (MAM), S1R can function at both the cellular and intercellular levels. Intracellularly, they act as chaperones, facilitating the correct folding of other proteins, regulating inositol-3-phosphate receptor function, stabilizing calcium signaling between the ER and mitochondria, and improving MAM lipid dynamics and stability. It has been proposed that S1R may be responsible for the metabolic regulation of mitochondria. Additionally, S1R activation through interaction with ion channels triggers a shift in neuronal excitability [9–12].

In the nervous system, S1Rs are detected on neurons and in astrocytes, microglia, and oligodendrocytes. They exhibit high expression in the cerebellum, hippocampus, locus coeruleus, anterior cingulate gyrus, cerebral cortex, thalamus, and hypothalamus. These receptors play important roles in physiological processes within the central nervous system and in synaptic plasticity, and they regulate numerous neurotransmitter systems, including neurosteroids, noradrenaline, dopamine, serotonin, acetylcholine, and glutamic acid [10]. Changes in S1R function or expression may lead to various neurological and psychiatric disorders, including Alzheimer’s disease, Huntington’s disease, amyotrophic lateral sclerosis, mood disorders, anxiety, and schizophrenia [11, 12].

### The structure and role of S2R

S2R is an under-researched 18- to 21-kDa protein having four transmembrane domains with N and C terminals extending to the cytoplasm [3]. This receptor forms an integral component of the endoplasmic reticulum membrane known as transmembrane protein 97 (TMEM97), and also referred to as meningioma-associated protein (MAC30). TMEM97 plays roles in cholesterol homeostasis and sterol transport in Niemann-Pick disease type C1. It also participates in the regulation of the intracellular concentration of calcium ions [13].

There are no currently known endogenous S2R ligands. The exogenous ligands include analogs of indole (ibogaine), tropane and granatane (BIMU-1, SW107, SW116, and SW120), cyclohexyl-piperazine (PB28 and F281), and 6,7-dimethoxytetrahydroisoquinoline (RHM-4, [^18^F] ISO-1, and [^125^ l] ISO-2) [14] (Table 2)*.*

**Table 2:** Agonists and antagonist S2R

Agonist	Antagonist
Haloperidol	Roluperidone (MIN-101)
DTG (1,3-di-o-tolylguanidine)	CT1812
CB-64D	RHM-4
CB-184	CM156 (3-(4-(4-cyclohexylpiperazin-1-yl)butyl)benzo[d]thiazole2(3H)-thione)
PB221	SAS-0132
PB28	SM21
Siramesine	CT0093
SV119	CT0109
UKH-1114	AC927 (1-(2- phenethyl)piperidine oxalate)
WC-26	SN79 (6-acetyl-3-(4-(4-(4-florophenyl)piperazin-1-yl)butyl)benzo[d]oxazol2(3H)-one)

High S2R expression is found in proliferating cells, including neoplasm cells. This receptor plays an essential role in cell differentiation and survival, which has prompted investigations of compounds that act on S2R, to assess their roles in neoplasms [15]. S2R is present in both CNS and peripheral tissues and can be found in lysosomes, the endoplasmic reticulum, and in the cell membrane. This receptor interacts with various other proteins—including caspase-3/7, cyclin D1, PARP-1, and EGFR—and is involved in the mobilization of ions (K^+^ and Ca^2+^) [13, 16, 17]. It has also been suggested that S2R may play important roles in neuroprotection and cognitive disorders, and could represent a potential target for the treatment of brain diseases [18].

### Sigma receptors and disorders of the nervous system

#### Alzheimer’s disease

Alzheimer’s disease (AD) is the most common cause of dementia, accounting for around 50–75% of all dementia cases. The etiopathogenesis of AD remains unknown. Neuron death occurs due to the deposition of pathological proteins in the brain—mainly the β-amyloid and the hyperphosphorylated *tau* proteins. AD is characterized by senile plaques and neurofibrillary tangles, which lead to disturbances in neuron functions, followed by neuron death and generalized brain atrophy. The loss of neurons leads to the clinical symptoms of AD. Cholinergic neurons are the most affected, while serotonergic, noradrenergic, and glutaminergic neurons disappear to lesser extents. Clinically, AD manifests with impaired memory and other cognitive functions. Behavioral and mood disorders are also frequent, which can make it impossible for the patient to independently function in everyday life [19].

In some cases, Alzheimer’s disease is genetic. Autosomal dominant AD results from mutations in the genes for the precursor proteins of amyloid, presenilin 1, and presenilin 2. Such mutations account for > 50% of early-onset familial AD, i.e., cases occurring before 65 years of age. However, this form constitutes less than 5% of all cases of AD. The majority of AD cases are sporadic and familial forms that occur after the age of 65. This population more commonly exhibits polymorphism of the ε4 allele in the gene for apolipoprotein E, which significantly increases the risk of AD development.

The senile plaques found in AD are mainly composed of β-amyloid (Aβ) deposits. Aβ is intracellularly produced in the MAM domain and may affect the functioning of the ER and MAM [20]. Considering the importance of S1R in MAM domains, it is not surprising that S1R polymorphisms increase the risk of AD [21]. Huang et al. [22] observed that certain combinations of various S1R and apolipoprotein (APOE) genotypes increase the risk of AD development.

Postmortem studies of AD patients reveal a decreased S1R density in the brain tissue; however, the reason for this change remains unclear [23]. Research has suggested that S1R plays a neuroprotective role in AD due to mechanisms, such as intracellular calcium regulation, anti-apoptotic effects, and prevention of oxidative stress [24]. Studies in animals demonstrate that S1R agonists improve memory processes through various mechanisms. In rat cortical cell cultures, T817MA can increase neurite growth and prevent sodium nitroprusside-induced cell damage, while administration of the S1R antagonist BD1047 can inhibit these processes [25]. Another selective S1R modulator, ( ±)-2-(3-chlorophenyl)-3,3,5,5-tetramethyl-2-oxazaphosphinate (OZP002), also exhibits neuroprotective effects in both genetic and pharmacological models of AD. OZP002 reportedly potentiates the antidepressant effect of another S1R agonist (igmesin) and prevents cognitive deficits in animal memory tests (the Y-maze and passive avoidance tests). Moreover, this effect was inhibited after administration of the S1R antagonist NE-100 [26]. Another compound that shows high affinity for S1R, and lesser affinity for S2R, is pridopidine, which has been studied in various neurodegenerative diseases, including HD, PD, and AD. In addition to affecting sigma receptors, pridopidine also acts on dopamine, serotonin, and adrenergic receptors. In mouse hippocampal cultures, pridopidine protects dendritic spines from the toxicity of the Aβ42 oligomer. S1R knockout leads to destabilization of dendritic spines, and pridopidine administration has no protective effect in these animals [27].

Available studies also indicate that S1R interacts with presenilin 1 (PS1) and presenilin 2 (PS2). Many familial mutations that cause mutations in PS1 and PS2 interfere with Ca^2+^ release from the ER through PS1 and PS2 channels, thereby increasing the calcium concentration inside the ER [28]. In hippocampal cell cultures, pridopidine inhibits calcium hemostasis by reducing the luminal concentration of calcium. The elimination of PS1, PS2, and PS2/2 results in the loss of dendritic spines in the hippocampal neurons. As mentioned above, pridopidine can compensate and restore the functionality of dendritic spines in neurons with presenilin 1 and 2 knockout [27].

Currently, ongoing clinical trials are evaluating the effectiveness of S1R for AD treatment. Donepezil, a cholinesterase inhibitor commonly used in AD, also shows an affinity for S1R. In an experimentally induced mouse model of memory impairment, caused by intraventricular injection of Aβ25-35 peptide, the administration of donepezil in combination with S1R agonists (PRE-084 and AVANEX2-73) yields synergistic effects and improves impaired memory processes. In contrast, an antagonistic effect was observed when S1R agonists were co-administered with memantine [29].

One investigation that has evaluated the efficacy of an S1R agonist was a 52-week randomized phase 2 clinical trial in patients with mild-to-moderate AD receiving edonerpic malate (previously evaluated in preclinical studies as T-817MA). This drug exhibited good tolerability and safety; however, the studied patients exhibited no improvement in memory impairment [30].

Another phase 2 randomized clinical trial in patients with AD investigated the effectiveness of Neudexta^®^ (Jenson Pharmaceutical Services Limited), also referred to as dextromethorphan, which shows an affinity for S1R and quinidine. The results indicated that Neudexta^®^ administration reduced anxiety (agitation)/aggression in the studied patients, but did not affect their cognitive processes [31]. Due to the observed reduction of aggression in AD patients, the drug was advanced to phase 3 clinical trials, and those results have not yet been published [32].

AVANEX2-73, a tetrahydrofuran derivative developed by Anavex Life Sciences, is currently in phase 2b/3 clinical trials. Preclinical studies in a mouse AD model demonstrated that AVANEX2-73 has a protective effect towards mitochondria, prevents tau hyperphosphorylation, and creates β (1–42) amyloid [33]. AVANEX2-73 also blocks the amnestic effects of β-amyloid injections into mouse brains, which can be reversed by administration of the S1R antagonist BD1047. Interestingly, AVANEX2-73 reverses the amnestic effects of scopolamine, an antagonist of muscarinic receptors, suggesting that multiple different receptors may be involved in the neuroprotective effects of this drug [34]. The phase 2b/3 clinical trial is focused on how AVANEX2-73 administration affects people with AD with regards to cognitive functions, sleep, and behavioral and psychological disorders typical for AD patients, as well as in terms of the patients’ daily functioning, burden on caregivers, and quality of life. The results of this trial have not yet been published [35].

In addition to S1R receptors, S2R receptors also play important roles in CNS functionality. Rivastigmine, a drug used in AD, increases NGF activation in the TrkA receptor signaling pathway. Rivastigmine also potentiates NGF-induced neurite outgrowth and Akt and ERK1/2 phosphorylation, which are completely inhibited after administration of the TrkA antagonist GW-441756. Neurite growth is not blocked by the acetylcholine receptor antagonists scopolamine and hexamethonium. However, co-administration of S1R (NE-100) and S2R (SM21) receptor antagonists yielded complete inhibition of rivastigmine-induced neuronal growth. These studies prove that both S1R and S2R are involved in neurite outgrowth, and suggest that rivastigmine may enhance neuron repair mediated by these two receptors [36].

S2R is also involved in β-42 amyloid neurotoxicity, and S2R antagonists (CT0093 and CT0109) may prevent neurotoxicity [37]. A study in mice overexpressing amyloid precursor protein (APP) demonstrated that S2R ligands (SAS0132 and DKR 1051) could prevent neuronal degeneration and improve cognitive functions [38].

The preclinical experiments showing neuroprotective effects of S2R ligands provided the basis for further clinical trials. A randomized clinical trial in healthy volunteers proved that the S2R antagonist CT1812 is well tolerated when administered in either single or multiple doses. Cognitive functions did not change between treatment initiation and the end of therapy [39]. Due to the excellent tolerance of CT1812, a phase 2 clinical trial was conducted in patients with mild-to-moderate AD; the results have not yet been published [40].

It is clear that compounds acting on both S1R and S2R may represent a therapeutic option for AD patients. However, we cannot comment on their effectiveness and safety until the research results are published.

#### Parkinson’s disease

Parkinson’s disease (PD) is a slowly progressing degenerative disease of the CNS, resulting from the degeneration of dopaminergic neurons and a dopamine deficiency in the substantia nigra and striatum of the brain. Some people with PD also exhibit protein aggregations, known as Lewy bodies, in the bodies of surviving neurons. Deficiency of dopaminergic neurons leads to motor disorders (motor slow-down, muscle tremors, and muscle stiffness) and mental disorders (depression and impaired cognitive processes). Patients with PD also exhibit irregularities in mitochondrial function, e.g., lowering the respiratory chain and oxidative and nitrative stress [41].

S1Rs have been found in both the substantia nigra and the striatum, and their numbers can be significantly reduced in PD patients [42]. S1R-knockout mice exhibit age-related motor abnormalities and degeneration of dopaminergic neurons, possibly resulting from the aggregation and phosphorylation of α-synuclein, which forms an abnormal structure due to oxidative stress and proteosome dysfunction [43]. S1R agonists have been proven effective in experimental PD models. In mice with a unilateral striatal hydroxydopamine lesion, Francardo et al. [44] demonstrated that chronic administration of the S1R agonist PRE-084 significantly improved motor functions of the animals that were promptly treated. Agonist administration was associated with increased striatal dopaminergic innervation and neuronal survival in the substantia nigra, and increased monoamine (dopamine and serotonin) levels and activities of neurotrophic factors (BDNF and GDNF). In another study, mice with experimentally triggered PD were given the S1R agonist pridopidine. The results proved that animals receiving the lower dose of pridopidine exhibited improved forelimb function and abolition of the ipsilateral rotational bias. These beneficial effects were not observed in mice with an experimentally induced striatal lesion in the absence of S1R [45]. An experiment in macaques with PD proved that pridopidine can reduce 3,4-dihydroxyphenylalanine-induced dyskinesia. Furthermore, pridopidine at doses that did not prevent dyskinesia still resulted in > 80% saturation of S1R receptors. When an effective amount was administered, they observed interactions with the α_2C_, dopamine D_3_, and serotonergic 5HT_1A_ receptors, suggesting that its binding only to the S1R may be insufficient to induce anti-Parkinsonian effects and that S1R may amplify the effects of these receptors [46].

Amantadine is a drug approved for PD treatment, which affects cholinergic and glutamatergic transmission. It also exhibits affinity for S1R receptors and may enhance G-protein activation in response to dopamine- and bradykinin-induced intracellular calcium mobilization, which can be reversed using the S1R antagonist BD1047 [47].

To date, the research on S1R agonists in PD has primarily involved preclinical studies. In September of 2020, researchers completed the second phase of a randomized clinical trial evaluating ANAVEX2-73 (S1R agonist) effectiveness in patients with PD with concomitant cognitive impairment. However, the results of this study have not yet been published, and thus the effectiveness of this drug remains unknown [48]. Similarly, phase 2 of a clinical trial evaluating the influence of pridopidine administration on the incidence of L-DOPA-induced dyskinesias in PD patients was completed in 2020, but no information is yet available regarding the effectiveness of this drug [49].

#### Amyotrophic lateral sclerosis

Amyotrophic lateral sclerosis (ALS) is an incurable fatal neurodegenerative disease characterized by progressive damage to motor neurons in the spinal cord and brain, leading to progressive muscle wasting. ALS-related disorders are caused by the intracellular accumulation of mutated and misfolded proteins. The best-known mutation, occurring in 20% of inherited forms of ALS, is the SuperOxide Dismutase 1 (SOD1) mutation [50]. S1R is strongly expressed in motor neurons [51]. Examination of motor neurons has revealed high S1R concentrations in the endoplasmic reticulum membrane, approximately 10 nm from the membrane of M_2_ muscarinic cholinergic neurons, as well as voltage-gated K^+^ channels (Kv2.1) and K^+^ channels activated with Ca^2^ (SK) [52]. S1Rs have also been found in the membrane of the mitochondrial endoplasmic reticulum, where they modulate the transfer of calcium from the ER to the mitochondria via the inositol triphosphate receptor.

In an experiment performed using S1R-knockout mice, observations included locomotor deficits, axonal degeneration, and loss of motor neurons [53]. Research in mice with mutation of the SOD1 gene proved that chronic use of the S1R agonist PRE-084 exerted neuroprotective action against this change (in SOD1), significantly improved the motor functions of the tested animals, and extended their survival by 15% [54]. Another study used a different S1R agonist, SA4503, and demonstrated increased survival of the tested mice but not improved locomotor function [55]. Other research has demonstrated that administration of the selective SIR agonist pridopidine reduced the aggregation of mutant SOD1, and improved motor neuron function [56]. In all of these studies, the beneficial effects of S1R agonists have been associated with the activation of signaling pathways, including protein kinase C, AKT, or ERK. Unfortunately, none of the studies have assessed the functions of MAM.

The possible roles of sigma receptors in other forms of ALS remain unknown. The research conducted to date has focused on familial forms involving the SOD1 mutation, which account for approximately 20% of all family cases, and < 2% of all ALS forms [57, 58]. Couly et al. [59] recently examined Drosophila with a genetically altered S1R and demonstrated that flies expressing the S1R^E102Q 108^ mutation (which is observed in ALS) exhibited disorders of eye development and altered mobility. These effects were accompanied by abnormal mitochondrial fragmentation, decreased ATP concentration, and greater “fatigue” of the neuromuscular junction during high energy demand. The authors proved that the S1R mutation leads to ALS and that increasing S1R density exerts a protective effect on neurons with the altered TDP43 protein.

The beneficial effects of S1R agonists in SLA may warrant investigations for effective medications that could act through this receptor. Smith et al. [60] published the results of a clinical trial in which ALS patients received Neudexta, a combination drug containing dextromethorphan and quinidine. Dextromethorphan is a medicine with a multidirectional mechanism of action, including S1R agonism, NMDA antagonism, and serotonin reuptake transporter affinity. The addition of quinidine to a multi-ion channel drug is intended to reduce the metabolism of dextromethorphan. In a study of 60 ALS patients, 70-day administration of Neudexta improved bulbar functions (speech disorders, salivation, and swallowing). A subsequent study to assess the efficacy of pridopidine in ALS has already been registered, but patient recruitment has not yet started [61]. The present evidence clearly supports the need for further studies to assess the effectiveness of S1R agonists in ALS.

#### Huntington’s disease

Huntington’s Disease (*HD*) is a genetic CNS disease. It is caused by mutations of the IT15 gene—which encodes the huntingtin protein—that increase the number of repeating sequences of CAG nucleotides. Healthy people have ≤ 35 CAG repeats. The presence of ≥ 36 CAG repeats is associated with the formation of abnormal huntingtin, which builds up in the neuron and causes damage, eventually completely eradicating it. Neuron destruction occurs as a result of oxidative stress, glial reactivity, altered intracellular signaling, impaired axonal transport, abnormal calcium regulation related to oxidative stress, excitotoxicity, and loss of synapses. The main symptoms of HD include movement disorders, progressive memory impairment, personality disorders, and mental disorders [11, 62].

The role of S1R in HD has been demonstrated based on cellular models. Hyrskyluoto et al. [63] showed a reduced number of S1R receptors in PC6.3 cell lines containing 120 glutamine repeats (120Q-huntingtin). Moreover, in this cell line containing mutant huntingtin proteins, administration of the S1R agonist PRE084 counteracted the damaging processes and increased neuron survival. It is possible that the neuroprotective effect of this S1R agonist was related to the normalization of the average concentrations of calpastatin and NF-κB-p65 in cells with huntingtin overexpression, yielding increased amounts of cellular antioxidants and decreased reactive oxygen species (ROS). The authors suggest that PRE084 administration increases S1R receptors by modulating post-transcriptional factors [63]. Another study demonstrated increased numbers of S1Rs in cell nuclei within the brains of patients with polyglutamine diseases, including HD, spinocerebellar ataxia type 1–3, and dentatorubral-pallidoluysian atrophy (DRPLA) [64]. S1R is sequestered in the nucleus through the actions of a specific exportin 1 inhibitor (leptomycin B) and the p62 protein. This investigation also demonstrated that thapsigargin, a non-competitive inhibitor of sarco-endoplasmic Ca^2+^-ATPase (SERCA), can cause S1R migration in the nucleus [64]. It is assumed that S1R can move between the nucleus and the cytoplasm, which may be related to the removal of nuclear inclusions containing the mutant huntingtin protein [11]. As mentioned earlier, S1Rs are chaperones that participate in the correct folding of proteins and the degradation of misfolded compounds. Miki et al. [65] suggest that S1R may be involved in the degradation of abnormal proteins from cell nuclei.

Pridopidine has been studied in HD patients, in both preclinical and clinical trials. It is an antagonist of the D_2_ receptor and an agonist of S1R, with its affinity for S1R being over 100 times higher than that for the D_2_ receptor [66]. Pridopidine also shows an affinity for α_2A/C_ adrenergic, serotoninergic (5HT_1A_ and HT_2A_), and histamine H_3_ receptors [11]. Squitieri et al. [67] investigated the effects of pridopidine administration in R6/2 mice with experimentally induced HD, finding that the drug improved motor parameters in both the open field test and the horizontal ladder test. The beneficial effects of pridopidine were related to the anti-apoptotic effect, restoration of a normal pERK1/2 concentration in the striatum, and increased expressions of neurostimulative and survival-promoting particles, i.e., striatal brain-derived neurotrophic factor (BDNF) and DARPP32 (cAMP-regulated neuronal phosphoprotein). It has also been reported that pridopidine administration reduces the concentration of mutant huntingtin in the striatum [67]. Ryskamp et al. [68] observed that in mice with experimentally induced HD, the beneficial effects of pridopidine may be associated with inhibition of dendritic spine loss. Moreover, loss of this protective effect has been observed in neurons lacking S1R, and following administration of the S1R antagonist NE100 [69]. Pridopidine has also been shown to inhibit excessive calcium release from the ER, normalize calcium levels in the ER, and reduce the excess calcium influx to dendritic spines [68]. A recent study demonstrated that pridopidine improves the motor activity in YAC128 mice with experimentally induced HD, as well as reduces symptoms of anxiety and depression. However, the drug did not prevent atrophy of the striatum and corpus callosum [70].

The available data indicate that pridopidine can modify the course of HD and alleviate symptoms. Several clinical trials have been conducted to evaluate the efficacy and safety of pridopidine in HD patients. The results indicate that this drug is well tolerated (Table 3); however, further research is required to assess the effectiveness of the therapy.

**Table 3:** Clinical trials evaluating the efficacy and safety of pridopidine in HD patients

Publication	Study	Pridopidine dose/patients number/treatment time	Results
Lundin et al. (2010) ACR16C007 [71]	Randomized, double-blind, placebo-controlled	50 mg/d (*n* = 48), Placebo (*n* = 30) 4 weeks	Compared to placebo, pridopidine doesn’t affect cognitive functions, involuntary movements, sleep disturbance, and depression. Pridopidine improves motor skills in patients, whose mMS was higher than 10 (vs placebo). In the group of patients who gets pridopidine, the improvement of cognitive functions was observed after 4 weeks of treatment compared to output value
de Yebenes et al. (2011) NCT00665223 [72]	Phase 3, randomised, double-blind, placebo-controlled trial	45 mg/d (*n* = 148), 90 mg/d (*n* = 145), Placebo (*n* = 144) 26 weeks	In the group of patients who gets 90 mg pridopidine statistically improvement in mMS score was observed after 26 weeks of treatment compared to placebo group. Other examined parameters hasn’t changed in group of patients who gets pridopidine
Huntington Study Group HART Investigators (2013) NCT00724048 [73]	Randomized, double-blind, placebo-controlled trial (HURT)	10 mg/d, 22.5 mg/d, 45 mg/d –for 4 weeks, 20 mg/d (*n* = 56) 45 mg/d (*n* = 55) 90 mg/d (*n* = 58) Placebo (*n* = 58) for 12 weeks	Any analized/examined dose of drug hasn’t improved statistically mMS score compared to placebo after 12 weeks of treatment. Despite that, the improvement of UHDRS-TMS score (p = 0.04 vs placebo) in patients, who gets 90 mg/d was observed
Esmaeilzadeh et al. (2011) [74]	Open study	45 mg/d for 1st week, 90 mg/d for 2nd week, (*n* = 8)	Increased metabolic activity in precuneus and dorsomedial nucleus of thalamus was observed in FDG PET
McGarry et al. (2017) NCT01306929 [75]	Multicenter open-label, randomized, placebo-controlled, dose-ranging, parallel-group study (OPEN-HART)	90 mg/d (*n* = 118) 36 months	TMS declined according to natural course of HD in last 36 months (lack of improvement after pridopidine). Major patients (*n* = 104; 89%) reported at least 1 side effect. In 75% patients side effect was mild or moderate. Declining frequency of AE occurrence was observed during the treatment
McGarry et al. (2020) [76]	Open-label extension of HART, a randomized, double-blind, placebo-controlled study	Continuation of the study OPEN_HART to 48 and 60 months 90 mg/d (*n* = 40) at 48 months and 90 mg/d (60 months)	Pridopidine appeared to be safe and well-tolerated drug after 60 months of treatment. Exploratory analysis showed that averaged decrease of TFC after 60 months was smaller in group of patients who gets pridopidine (0.4 point/year) in compare to control group (1 point/year)
Reilmann et al. (2019) NCT02006472 [77]	Phase 2, randomised, placebo-controlled, multicentre, dose-ranging study (PRIDE-HD)	45 mg/d (*n* = 81) 67.5 mg/d (*n* = 82) 90 mg/d (*n* = 81) 112.5 mg/d (*n* = 82) Placebo (*n* = 82) 26 tygodni	Any analyzed/examined dose of drug hasn’t improved UHDRS-TMS score. Most common side effects in patients who get pridopidine: insomnia, diarrhea, nausea, dizziness
Grachev et al. (2020) NCT03019289 [78]	Single-dose, open-label, adaptive design PET study	[^18^F] fluspidine PET–male volunteers (*n* = 11; 0.5 mg, 1 mg, 5 mg, 22.5 mg, 45 mg, 90 mg–single dose) and man with HD (*n* = 3, 90 mg–single dose) [^18^F] fallypride PET–male volunteers (*n* = 4; 90 mg–single dose)	After pridopidine administration in 22.5–90 mg dose affinity to S1R was 87–91%. At the dose 90 mg affinity to D_2_ and D_3_ was only 3%

*mMS* modified motor score, *UHDRS* Unified Huntington’s disease rating scale, *UHDRS-TMS* Unified Huntington’s disease rating scale-total motor score, *FDG PET* [^18^F]Fluorodeoxyglucose positron emission tomographic, *TMS* total motor score, *TFC* functional capacity score

#### Stroke

In vitro and preclinical studies prove that sigma receptors ligands exert neuroprotective activity. A number of pathological conditions, including stroke and traumatic damage, involve a long-lasting release of glutamate and a high influx of calcium into the cell, resulting in toxic effects on the cell and ultimately in cell death. This phenomenon is called excitotoxicity, and NMDA receptors play a special role in its occurrence. Sigma receptor ligands have reportedly reduced neurotoxicity in primary neuronal cultures and in ischemic stroke models [79, 80]. Ajmo et al. [81] found that the administration of DTG (1,2-di-tolylguanidine) 24 h after ischemic stroke increases neuronal survival in rats. Likely mechanisms underlying this neuroprotective action include a reduction of glutamate release, decreased levels of NMDA receptor activation or expression, and an altered intracellular calcium concentration [82]. The protective role of S1R was confirmed by Morihara et al. [83], who showed that a novel S1R agonist (Comp-AD) reduced the ischemic stroke zone in mice. This effect was related to S1R upregulation and the reduction of endoplasmic reticulum stress.

Sánchez-Blázquez et al. [84] investigated the effect of an S1R antagonist, rather than an agonist, in an animal model of cerebral ischemia. Mice were subjected to occlusion of the central cerebral artery, and then the volume of the brain affected by ischemic stroke and the neurological losses were determined at various time intervals following artery closure. Administration of the S1R antagonist (E-52862/MR309) significantly reduced the cerebral infarction size and the neurological deficits. This neuroprotective effect was observed when the compound was administered 5 h before surgery, as well as at 3 h after surgery. Additionally, in the infarct-affected cortex, there were significant decreases in metalloproteinase 9 and astrocyte gliosis.

In 2011, Ruscher et al. [85] reported increased S1R expression in the peri-infarct areas of rats following permanent occlusion of the middle cerebral artery. Administration of the S1R agonist SA4503 at two days after the onset of the infarction restored the lost sensorimotor function, without reducing the total infarct area. Following S1R stimulation in peri-infarct areas, membrane rafts exhibited decreased concentrations of the synaptic proteins neurabine and neurexin. The authors concluded that S1R activation triggers regeneration following a stroke.

Since preclinical studies demonstrated a beneficial role of SA4503, this compound was tested in patients after ischemic stroke. In a randomized phase 2 clinical trial, 60 patients who had experienced a stroke were administered cutamesine (SA4503) for 28 days and were then monitored for the next 28 days after drug discontinuation. Cutamesine was started on the 3rd day after the stroke. The drug was safe and well-tolerated by the patients. However, no significant improvements were observed in the primary end-points: changes in the National Institutes of Health Stroke Scale (NIHS), Modified Rankin’s score, and Barthel’s score. The post-hoc analysis revealed that patients with moderate (NIHS ≥ 7) and severe (NIHS ≥ 10) stroke, within the subgroup of patients who received a higher dose of cutamesine, exhibited statistically significant improvement in the NIHS scale compared to the placebo group [86]. To date, no other clinical trials have been conducted with S1R ligands in patients with stroke. Additional studies are needed to assess the roles of the neuroprotective effects of sigma receptor agonists and antagonists in patients after stroke.

#### Epilepsy

Epilepsy affects approximately 0.5–1% of the world’s population. This disease manifests as convulsive or non-convulsive, and the seizures result from abnormal neuronal discharges. Despite the vast number of antiepileptic drugs available, many patients do not achieve satisfactory seizure control [87]. Thus, there remains a need to search for new drugs with new mechanisms of action. One possible therapeutic option is the use of sigma receptor ligands.

Experimental studies have demonstrated that sigma receptor ligands have anticonvulsant activity. In animal studies, S1R agonists (dextromethorphan, dimemorfan, and pentoxyverine) have been shown to prevent kainic acid-induced seizures [88, 89]. In experiments involving cocaine-induced seizures, anticonvulsant effects have been exerted by S1R antagonists (rimicazole analogues, BD-1008, and AC-927 derivatives) [90–92]. Another study revealed that the antagonist NE-100 exerted a proconvulsive effect in pentylenetetrazole (PTZ)-induced convulsions, while the agonist PRE-084 had no effect on seizures stimulated by PTZ and bicuculine (BIC). On the other hand, the allosteric S1R modulator ER1 showed anticonvulsant activity in clonic and tonic seizures induced by PTZ and BIC [93].

Other S1R modulators also exhibit anticonvulsant activity, including phenytoin, which is well known as an anti-epileptic drug. Other such modulators include SKF83959 and SOMCL-668, which have exhibited anticonvulsant activity in convulsions induced by PTZ and kainic acid [94].

To date, no clinical trials have been conducted to evaluate the role of sigma receptor ligands in epilepsy. Based on the effects of both S1R agonists and antagonists in various experimental models, further studies should be conducted to elucidate the role of sigma receptors in these anticonvulsant effects.

#### Multiple sclerosis

Multiple sclerosis (MS) is a chronic autoimmune and inflammatory demyelinating disease of the CNS, in which the myelin sheath is disrupted. MS is most frequently diagnosed in young adults, and often leads to severe neurological problems. Symptoms of this disease include movement, sensory, and cerebellar disorders, as well as visual disturbances, urological problems, and autonomic, psychiatric, and cognitive troubles [95].

Several studies have demonstrated the role of S1R ligands in myelin synthesis and oligodendroglial proliferation. Chechneva et al. [96] showed that the S1R agonist dextromethorphan (DM) inhibited experimentally induced autoimmune encephalitis in an animal model of MS. Additionally, Demerens et al. [97] noted that the S1R agonist eliprodil enhanced myelination in cultures of mouse cerebellar cells. Moreover, the S1R agonists AVANEX 2–73 and DM reportedly protected oligodendroglia (OL) and oligodendroglial precursors (OPC) against apoptosis and excitotoxicity. Avanex2-73 also increased the OPC proliferation index (by 46% compared to in untreated cultures), and this effect was inhibited by the S1R antagonist BD1047 [98, 99].

Currently available medications for MS slow the disease progression but do not promote repair and remyelination. It is possible that S1R agonists will be a new therapeutic option in MS patients; however, no studies presently support this hypothesis.

## Conclusion

Sigma receptors are an insufficiently understood group of receptors. To date, the conducted research has provided information regarding their structure, role in cellular functioning, and distribution in the body. Notably, it has been found that sigma receptors play a particularly significant role in the CNS. In the current article, we have described the use of sigma receptor ligands in a variety of neurological disorders. We presented the results of numerous preclinical and clinical studies and discussed the possible mechanisms underlying the roles of these receptors in the development of CNS disorders.

Previous research has demonstrated the roles of sigma receptors in the pathophysiology of multiple CNS diseases and has shown that sigma receptor agonists and antagonists may be useful for the management of these diseases. Our present knowledge regarding the mechanisms of action of individual ligands remains insufficient, and further research is required. This is clear from the results of some preclinical studies, which have demonstrated the beneficial roles of both agonists and antagonists in the development of individual diseases. Research on sigma receptor ligands has uncovered numerous compounds showing an affinity for particular types of sigma receptors. These receptor ligands include compounds that are not presently applied in medicine, as well as drugs that are already used in various CNS diseases and in disorders affecting the peripheral system. However, additional research is required to elucidate the exact roles of the individual ligands of sigma receptors.

Undoubtedly, a better understanding of the roles of sigma receptors would make it easier to find physiological ligands. Elucidating the physiological foundations of the roles of sigma receptors, and finding natural neurotransmitters, would greatly expand our knowledge on this subject. Many already registered drugs with different modes of action have impacts on sigma receptors, although the influences have not been explained. A more precise understanding of the characteristics of these receptors will be highly important for assessing the effectiveness in various afflictions, and for determining the safety and the interactions of these drugs with other compounds.
